# Femoral artery pseudoaneurysm as a late complication of flexible intramedullary nailing for a diaphyseal femur fracture in a child: a case report

**DOI:** 10.1590/1677-5449.202501282

**Published:** 2026-03-30

**Authors:** Gabriel Bezerra Pereira, Pedro Raphael Rocha de Sousa, Armando Nicodemos Lucena Felinto, Mariana Almeida Sales, Jurandir Vieira Marques, Paulo Giordano Baima Colares

**Affiliations:** 1 Universidade Estadual do Ceará – UECE, Fortaleza, CE, Brasil.; 2 Hospital Instituto Dr. José Frota, Fortaleza, CE, Brasil.

**Keywords:** pseudoaneurysm, femoral fracture, intramedullary fracture fixation, orthopedics, case reports

## Abstract

Flexible intramedullary nails are a safe, effective, and low-morbidity option for the treatment of diaphyseal femur fractures in children. They generally provide acceptable results regarding bone consolidation and clinical stabilization. However, late vascular events may occur. In this report, we describe the case of a male patient who underwent osteosynthesis with flexible nails for a femur fracture at age 9 and 5 years later developed local pain and swelling, leading to detection of a left femoral artery pseudoaneurysm. Subsequently, surgical correction with revascularization using an ipsilateral great saphenous vein was performed, with no further complications and an adequate clinical outcome. This case emphasizes the importance of long-term surveillance in orthopedic follow-up, particularly in the context of vascular injuries after surgical interventions.

## INTRODUCTION

Trauma is the leading cause of morbidity and mortality among pediatric patients. Femoral fractures account for 1.4 to 2% of all fractures of the immature skeleton, with bimodal presentation, peaking initially from 2 to 4 years of age and again during adolescence, most often involving the metadiaphyseal section.^1,2^ They are generally caused by high energy traumas, such as traffic accidents, and may need surgical treatment, depending on patient age and the characteristics of the fracture.^3^

For some years, flexible intramedullary nailing has become the surgical treatment of choice for diaphyseal femur fractures in children aged 5 to 11 years, ideally for patients with body weight below 50 kg.^4^ Titanium nails can be inserted using a retrograde technique via 2 cm skin incisions in the medial and lateral areas of the distal third of the thigh, at the level of the distal growth plate of the femur. This technique offers greater stability, with minimal tissue damage, adequate return of function, and low rates of serious complications.^1,3^ The most common complications are related to localized peri-implant soft tissue damage, such as pain and discomfort caused by prominence at the point of nail insertion.^5^

Vascular complications are rare forms of post-surgical progression. A pseudoaneurysm is an injury to the vessel wall, with blood leakage to the extravascular space and a potential risk of exsanguination and hemorrhagic shock, but they tend to be contained by structures around the lesion. When pseudoaneurysms are accompanied by symptoms, patients generally present progressive pain, claudication, or local swelling.^6^ Moreover, it is the presence of symptoms that arouses suspicion of a pseudoaneurysm, which thus demands an active investigation of any complaints of persistent pain during follow-up, which can be supplemented with imaging exams, such as angiotomography or Doppler ultrasound.^7^

This paper describes a rare case of pseudoaneurysm of the deep femoral artery as a late complication of osteosynthesis with flexible intramedullary nails in a pediatric patient with a diaphyseal femur fracture. There are few similar reports in the literature, underscoring the relevance of the case for orthopedic practice.

## CASE REPORT

A 14-year-old, previously healthy male patient with no family history of vascular or hematological diseases had suffered a diaphyseal fracture to the left femur at the age of 8 years, after falling from a wall onto his left leg. At the time, the patient had been assessed and treated with osteosynthesis using flexible titanium intramedullary nails because he met the criteria for this treatment. After the procedure, there were no immediate complications and the patient recovered clinical stability, with bone consolidation as expected, and was followed-up in outpatients to screen for complications.

Five years after the procedure, the patient returned to the clinic, describing progressive, insidious pain in the left thigh, unrelated to any de novo trauma. On physical examination, there was localized swelling and elevated sensitivity in the distal medial area of the left thigh, with a hard nodule measuring 4 x 3 cm, presence of rebound on palpation and percussion, and no obvious signs of inflammation or changes affecting gait.

**Figure 1:**
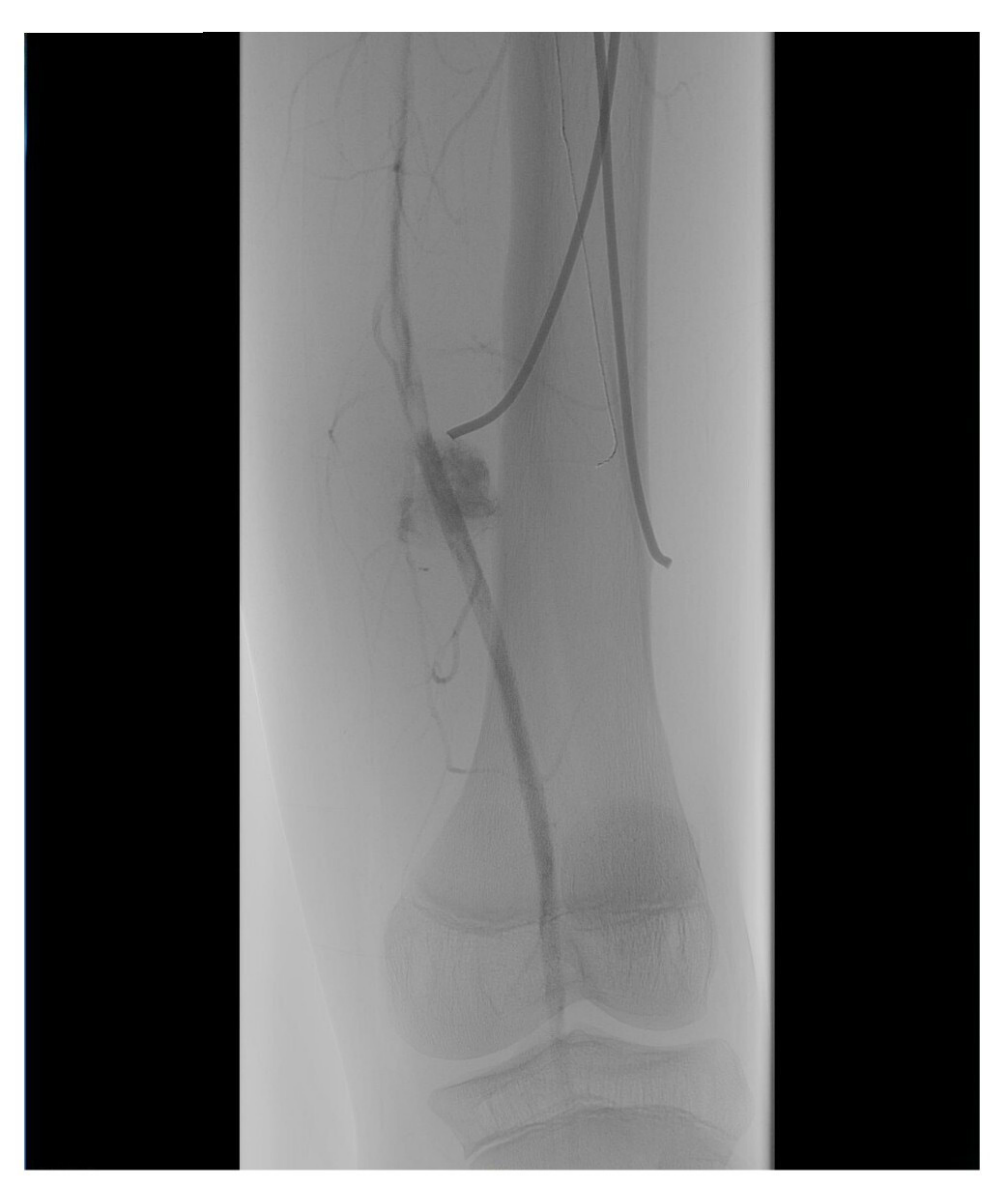
Arteriography of the left lower limb, showing saccular dilatation characteristic of a femoral artery pseudoaneurysm, adjacent to the tip of the medial nail (1/2). Source: Archives of the Vascular Surgery Department, Hospital Instituto Dr. José Frota.

**Figure 2:**
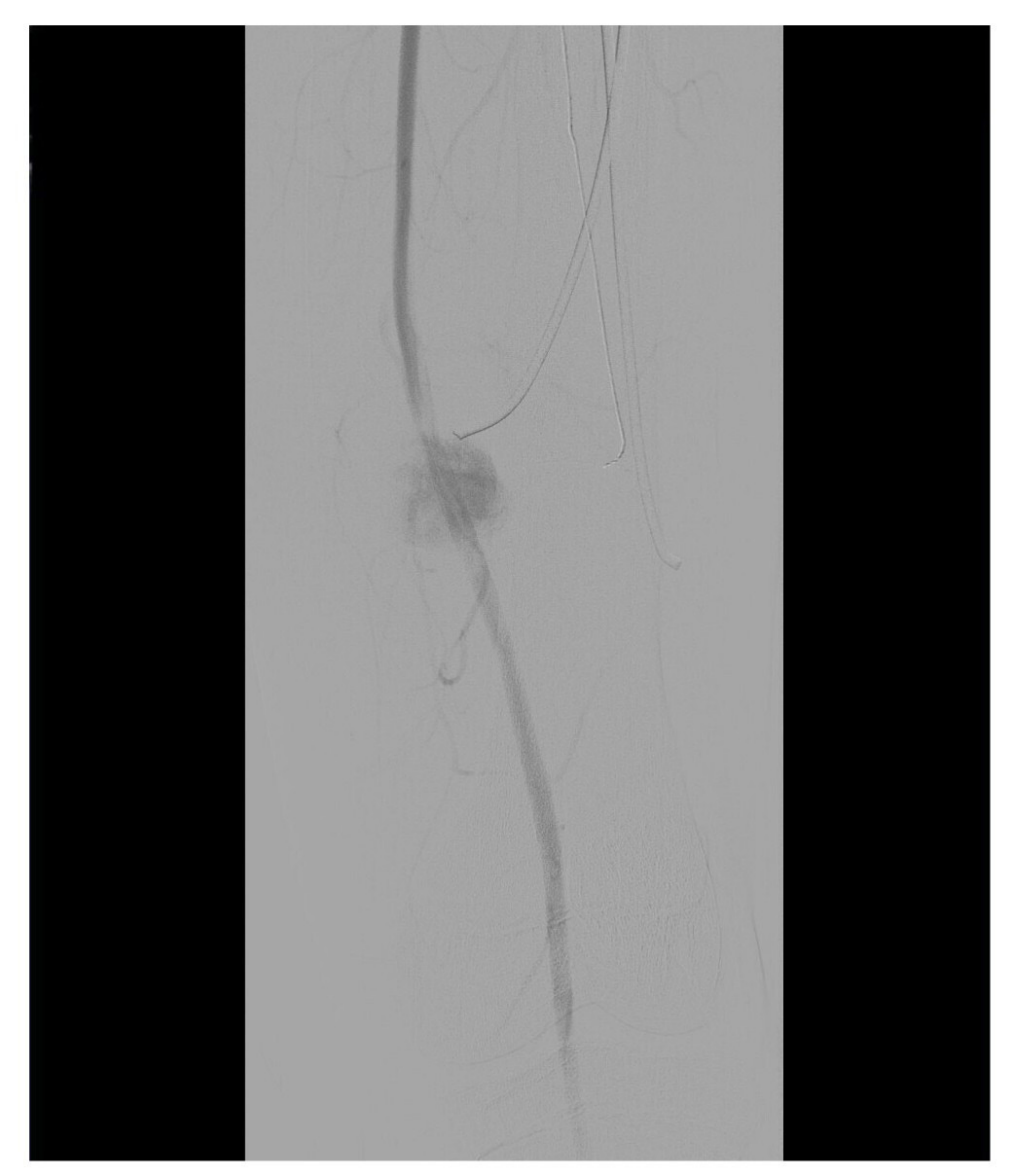
Arteriography of the left lower limb, showing saccular dilatation characteristic of a femoral artery pseudoaneurysm, adjacent to the tip of the medial nail (2/2). Source: Archives of the Vascular Surgery Department, Hospital Instituto Dr. José Frota.

In view of this presentation, clinical investigation was initiated in conjunction with the vascular surgery team and imaging exams were ordered to support diagnosis. Doppler ultrasound of the left thigh found no signs of deep venous thrombosis, but showed evidence suggestive of aneurysmal dilatation of the superficial femoral artery. Based on these findings, the patient was admitted to schedule surgery, when arterial angiography of the left lower limb was conducted, also yielding images suggestive of an aneurysm of the distal third of the left femoral artery, as illustrated in Figures 1 and 2.

The patient underwent resection of the pseudoaneurysm and vascular reconstruction with a femorofemoral bypass, using an ipsilateral great saphenous vein reverse graft. During the same procedure, the flexible nail positioned in the medial left femur was removed since it had probably caused the injury that culminated in the pseudoaneurysm. The lateral nail was not removed to avoid excessive operating time and surgical aggression. The procedure was completed with no complications and the patient recovered well postoperatively, with remission of pain and gradual recovery of full mobility.

**Figure 3:**
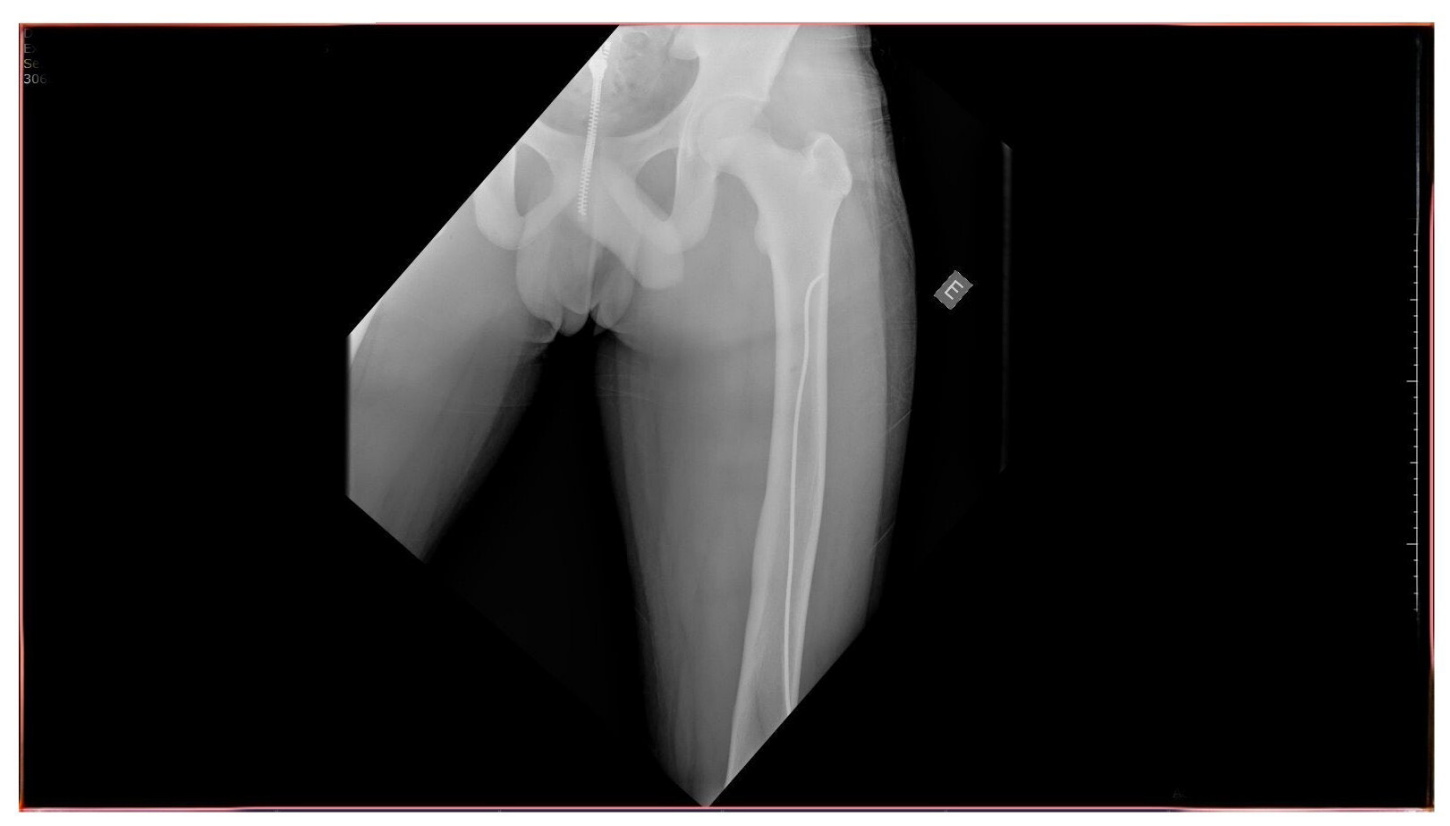
Control radiography conducted 8 months after the procedure showing good bone consolidation and the flexible nail in a normal position within the left femur (1/3) Source: Archives of the Vascular Surgery Department, Hospital Instituto Dr. José Frota.

**Figure 4:**
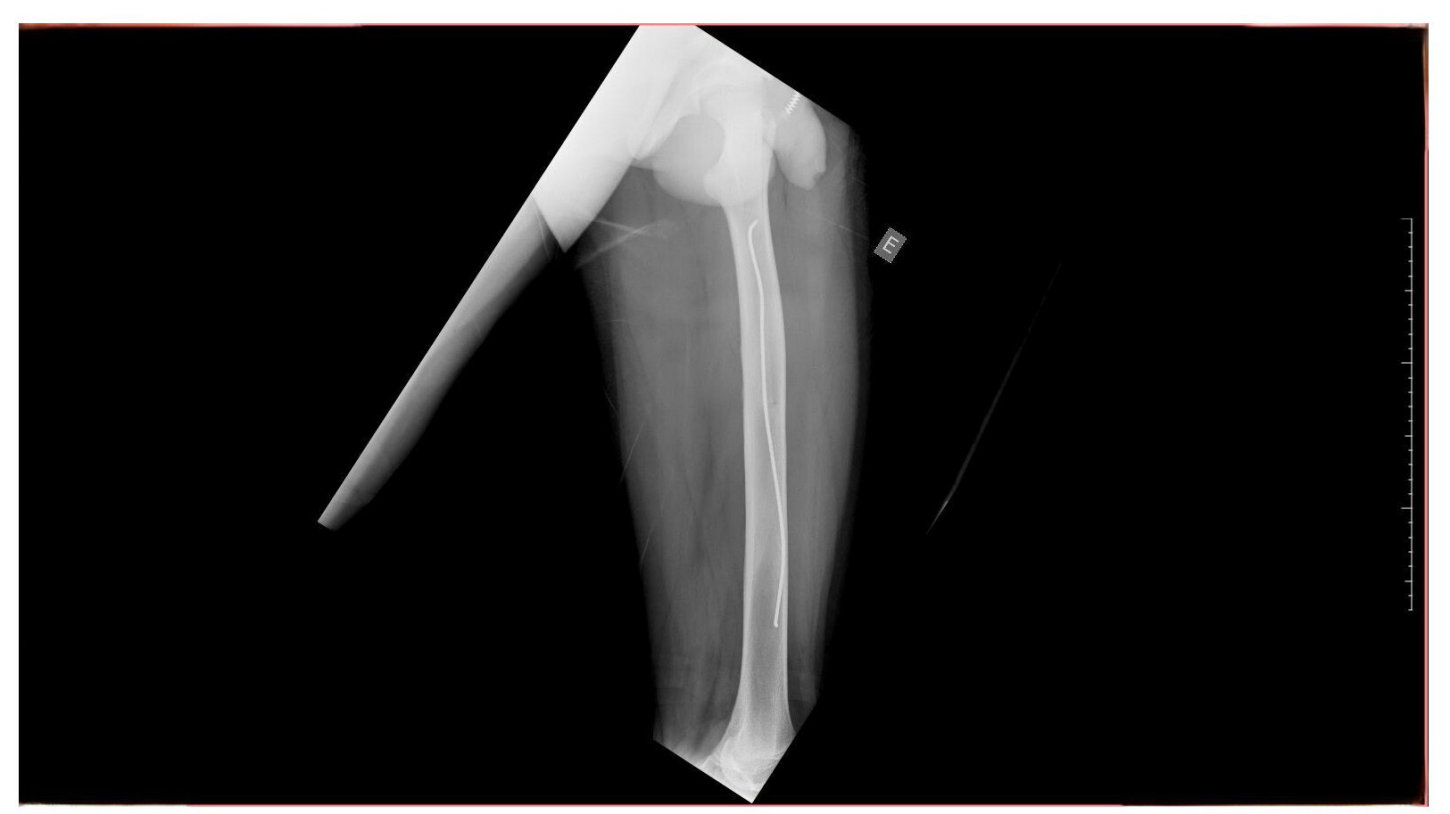
Control radiography conducted 8 months after the procedure showing good bone consolidation and the flexible nail in a normal position within the left femur (2/3). Source: Archives of the Vascular Surgery Department, Hospital Instituto Dr. José Frota.

**Figure 5:**
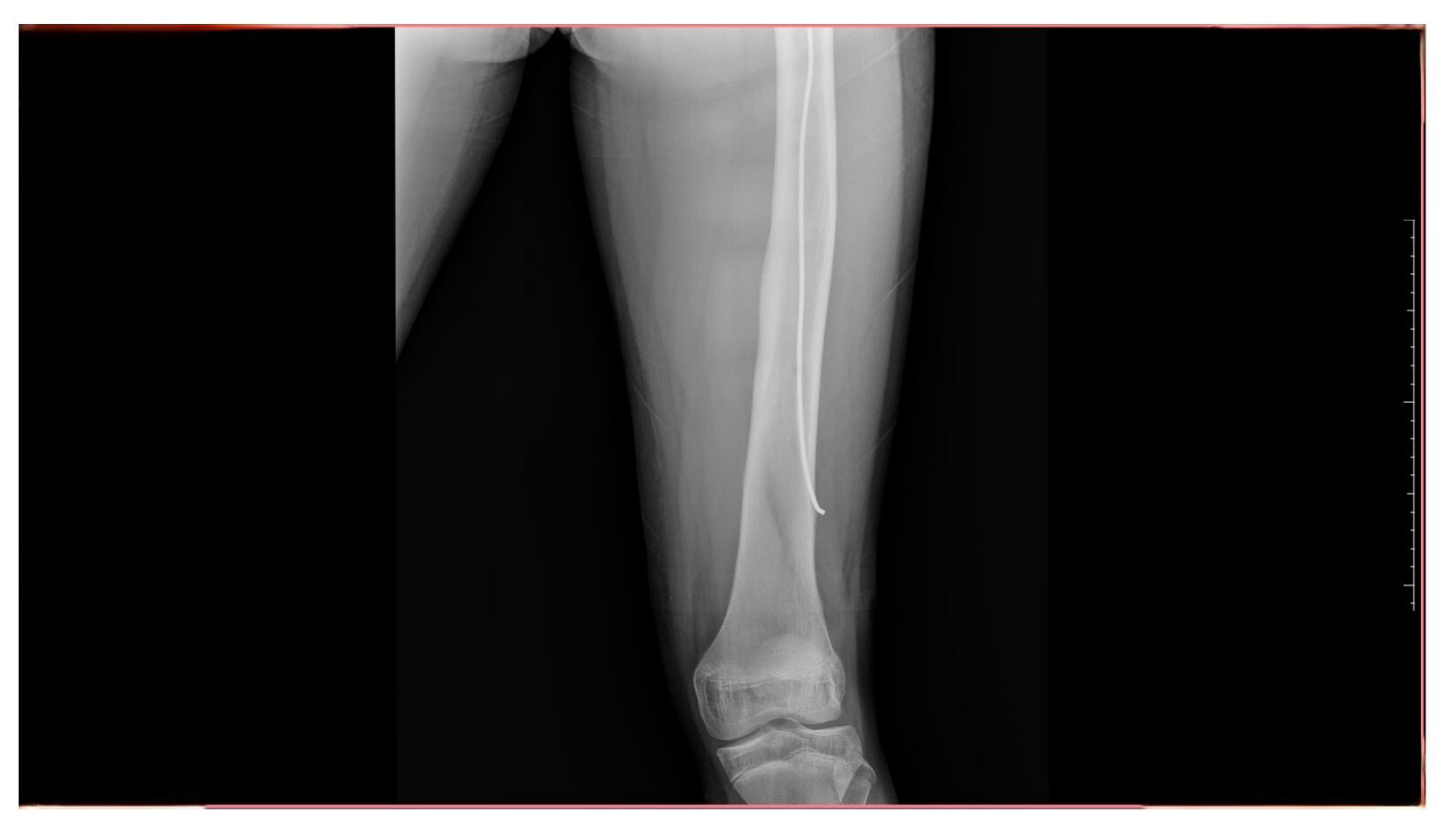
Control radiography conducted 8 months after the procedure showing good bone consolidation and the flexible nail in a normal position within the left femur (3/3). Source: Archives of the Vascular Surgery Department, Hospital Instituto Dr. José Frota.

In June 2025, 8 months after the last surgical intervention, the patient returned to the traumatology and orthopedics department, free from pain, with good bone consolidation and no new complications, with full and painless return to routine activities, ascertained during history taking. Physical examination found no physical deformities or edema and the patient demonstrated left knee joint movement with amplitude of 135º in flexion to 0º in extension. Control X-rays showed the lateral flexible nail in the normal position and no obvious changes, as illustrated in Figures 3, 4, and 5.

## DISCUSSION

Fractures involving the femoral diaphysis in children are often corrected surgically using flexible nails, especially in patients aged 5 to 11 years and with body weight of less than 50 kg.^4^ This surgical method can reduce the time spent in hospital and the rate of serious complications.^1^

Moreover, beyond the limitations inherent to the type of study presented, such as the impossibility of generalization of findings, it is also important to point out that adequate longitudinal surveillance is essential, regardless of the rarity of vascular complications. Formation of an arterial pseudoaneurysm as a late complication is especially rare. There are very few similar reports in the medical literature and those cases that have been described are generally associated with problems such as migration of implants, incorrect positioning of surgical hardware, or gradual and progressive vascular wear caused by friction.^8^

It is also important to stress that a pseudoaneurysm of the left femoral artery constitutes a serious threat to limb function and can cause compression of nerves and muscles, thrombosis, or even rupture with exsanguination. The surgical treatment option was the most appropriate in this case, in view of the progressive clinical manifestations, the risk of ischemic complications, and the images confirming presence of an aneurysm. Vascular reconstruction with a femorofemoral bypass using an ipsilateral great saphenous vein reversed graft proved to be an effective technique.^9^

This case also serves as a warning that vascular complications should be monitored as potential consequences of orthopedic surgery performed for osteosynthesis of fractures, including in pediatric patients. Appropriate follow-up with thorough investigation is essential to rule out such potentially severe complications.

This study was authorized by the Research Ethics Committee, under opinion number 7.492.055 and complies with the requirements of Resolution 466/2012 for protection of patient data and confidentiality.

## CONCLUSIONS

The incidence of vascular complications related to osteosynthesis is very low, but the present case report describing an arterial pseudoaneurysm that emerged 5 years after a surgical procedure in a pediatric patient illustrates the importance of appropriate longitudinal follow-up, with clinical suspicion, use of diagnostic imaging, and orthopedic and vascular surgeons working in conjunction for early identification of vascular events, which, while rare, can be clinically significant.

## DISPONIBILIDADE DE DADOS

Os dados que sustentam este estudo estão disponíveis mediante solicitação ao autor correspondente, GBP, para garantir a privacidade dos participantes.
