# Supplementary material for: Femoral artery pseudoaneurysm as a late complication of flexible intramedullary nailing for a diaphyseal femur fracture in a child: a case report
Source: J Vasc Bras. 2026 Mar 30;25:e20250128. doi: 10.1590/1677-5449.202501282 (PMC13078175; doi:10.1590/1677-5449.202501282)
Supplement: Translation

# Translation: Pseudoaneurisma em artéria femoral como complicação tardia de osteossíntese de fratura diafisária de fêmur em criança com haste intramedular flexível: relato de caso

**DOI:** 10.1590/1677-5449.202501281

**Authors:** Gabriel Bezerra Pereira, Pedro Raphael Rocha de Sousa, Armando Nicodemos Lucena Felinto, Mariana Almeida Sales, Jurandir Vieira Marques, Paulo Giordano Baima Colares

**Affiliations:** 1 Universidade Estadual do Ceará – UECE, Fortaleza, CE, Brasil.; 2 Hospital Instituto Dr. José Frota, Fortaleza, CE, Brasil.

**Keywords:** pseudoaneurisma, fraturas do fêmur, fixação intramedular de fraturas, ortopedia, relato de caso

## Abstract

As hastes intramedulares flexíveis são opções seguras, eficazes e de baixa morbidade para o tratamento de fraturas diafisárias do fêmur em crianças. Geralmente, proporcionam resultados aceitáveis no que diz respeito à consolidação óssea e à estabilização clínica. No entanto, pode haver alguns eventos vasculares tardios. Neste relato, descrevemos um caso de um paciente do sexo masculino que foi submetido a osteossíntese com hastes flexíveis em fêmur aos 9 anos e evoluiu, após 5 anos, com dor e abaulamento local, tendo sido detectado pseudoaneurisma de artéria femoral esquerda. Na sequência, foi realizada correção cirúrgica com revascularização através de veia safena magna ipsilateral, sem novas intercorrências e com adequada evolução clínica. O presente caso enfatiza a importância da vigilância a longo prazo no acompanhamento ortopédico, particularmente no contexto de lesões vasculares após intervenções cirúrgicas.

## INTRODUÇÃO

O trauma é a principal causa de morbimortalidade em pacientes na faixa etária pediátrica. As fraturas de fêmur somam 1,4 a 2% do total de fraturas no esqueleto imaturo e possuem apresentação bimodal entre os 2 e 4 anos e durante a adolescência, mais comumente em região metadiafisária^1,2^. Além disso, decorrem geralmente de traumas de alta energia, como acidentes automobilísticos, e podem necessitar de tratamento cirúrgico, a depender da idade do paciente e das características da fratura^3^.

Há alguns anos, o uso de hastes intramedulares flexíveis se tornou o tratamento cirúrgico de eleição para fraturas diafisárias de fêmur em crianças de 5 a 11 anos, idealmente com peso corporal abaixo de 50 kg^4^. As hastes feitas de titânio podem ser inseridas retrogradamente, através de incisões de pele de 2 cm nas regiões medial e lateral de terço distal da coxa, ao nível da placa de crescimento distal do fêmur. Essa técnica permite maior estabilidade, com mínima lesão tecidual, além de antecipar um retorno funcional adequado, com baixas taxas de complicações graves^1,3^. As complicações mais comuns se referem a danos locais em tecidos moles peri-implantares, como dor e desconforto por proeminência em sítio de inserção da haste^5^.

Complicações vasculares são formas raras de evolução pós-cirúrgica. O pseudoaneurisma é o termo que denota uma lesão em parede vascular, com saída de sangue para o meio extravascular, potencial risco para exsanguinação e choque hemorrágico, porém, costuma ser contido por estruturas ao redor da lesão. Quando o pseudoaneurisma ocorre acompanhado de sintomas, os pacientes geralmente apresentam dor progressiva, claudicação ou aumento de volume local^6^. Além disso, é a presença de sintomas que possibilita a suspeita do pseudoaneurisma, o que demanda investigação ativa de queixas álgicas persistentes durante o acompanhamento e que deve ser complementada por exames de imagem, como angiotomografia ou ultrassonografia com Doppler^7^.

Neste trabalho, relatamos um caso raro de pseudoaneurisma da artéria femoral profunda como complicação tardia de osteossíntese com hastes intramedulares flexíveis em paciente pediátrico que havia sofrido fratura diafisária de fêmur. Há poucos relatos semelhantes na literatura, o que ressalta a relevância do caso apresentado para a prática ortopédica.

## RELATO DO CASO

Paciente de 14 anos, do sexo masculino, previamente hígido, sem histórico familiar de doenças vasculares ou hematológicas, sofreu uma fratura diafisária de fêmur esquerdo aos 8 anos, após queda de muro de uma casa sobre sua perna esquerda. Na ocasião, o paciente foi avaliado e, posteriormente, recebeu indicação à osteossíntese por meio de hastes intramedulares flexíveis de titânio por preencher critérios para essa terapêutica. Após o procedimento, não houve complicações imediatas e o paciente evoluiu com estabilidade clínica e consolidação óssea conforme esperado, permanecendo em seguimento ambulatorial para rastreio de complicações.

Cinco anos após o procedimento, o paciente retornou ao ambulatório com relato de dor progressiva na coxa esquerda, de caráter insidiosa, sem novo trauma associado. Ao exame físico, apresentava abaulamento local e sensibilidade aumentada em topografia medial distal da coxa esquerda, com nódulo de característica endurecida de medidas 4 x 3 cm, presença de rechaço à palpação e à pulsação, sem sinais inflamatórios exuberantes ou alteração evidente da marcha.

Diante do quadro, foi iniciada investigação ambulatorial em conjunto com a equipe de cirurgia vascular, sendo solicitados exames de imagem para elucidar diagnóstico. O exame de ultrassom com Doppler de coxa esquerda não mostrou sinais de trombose venosa profunda, mas evidenciou imagem sugestiva de dilatação aneurismática da artéria femoral superficial. Com base nos achados, o paciente foi internado para programação cirúrgica, momento em que foi realizada angiografia arterial de membro inferior esquerdo, a qual também evidenciou imagem sugestiva de aneurisma em terço distal da artéria femoral esquerda, conforme ilustrado nas Figura 1 e 2.

**Figura 1:** Arteriografia de membro inferior esquerdo, evidenciando dilatação sacular característica de pseudoaneurisma em artéria femoral, adjacente à ponta da haste medial (1/2). Fonte: Arquivos do setor de Cirurgia Vascular do Hospital Instituto Dr. José Frota.

**Figura 2:** Arteriografia de membro inferior esquerdo, evidenciando dilatação sacular característica de pseudoaneurisma em artéria femoral, adjacente à ponta da haste medial (2/2). Fonte: Arquivos do setor de Cirurgia Vascular do Hospital Instituto Dr. José Frota.

O paciente foi submetido a ressecção de pseudoaneurisma e reconstrução vascular com ponte femoro-femoral, utilizando veia safena magna ipsilateral em posição reversa. No mesmo tempo cirúrgico, foi realizada retirada de uma das hastes flexíveis posicionadas medialmente ao fêmur esquerdo que provavelmente ocasionou a lesão, deflagrada em pseudoaneurisma. A retirada da haste lateral não foi realizada para evitar maior tempo e agressão cirúrgica. O procedimento não teve intercorrências e o paciente apresentou boa recuperação pós-operatória, com remissão da dor e retorno gradual à mobilidade plena.

Em junho de 2025, 8 meses após última resolução cirúrgica, o paciente retornou ambulatorialmente ao serviço de Traumato-Ortopedia, sem queixas álgicas, evoluindo com adequada consolidação óssea e sem novas complicações, com retorno completo e indolor às suas atividades cotidianas, investigadas em anamnese. No exame físico, não foram identificadas deformidades aparentes, instabilidade ou edemas, e o paciente demonstrou amplitude de movimento articular de joelho esquerdo com 135º em flexão e 0º em extensão. As radiografias de controle com presença de haste flexível lateral normoposicionada não mostraram alterações evidenciadas, conforme Figuras 3, 4 e 5.

**Figura 3:** Radiografia de controle realizada 8 meses após o procedimento que evidencia adequada consolidação óssea e uma haste flexível normoposicionada em fêmur esquerdo (1/3) Fonte: Arquivos do setor de Imagem do Hospital Instituto Dr. José Frota.

**Figura 4:** Radiografia de controle realizada 8 meses após o procedimento que evidencia adequada consolidação óssea e uma haste flexível normoposicionada em fêmur esquerdo (2/3). Fonte: Arquivos do setor de Imagem do Hospital Instituto Dr. José Frota.

**Figura 5:** Radiografia de controle realizada 8 meses após o procedimento que evidencia adequada consolidação óssea e uma haste flexível normoposicionada em fêmur esquerdo (3/3). Fonte: Arquivos do setor de Imagem do Hospital Instituto Dr. José Frota.

## DISCUSSÃO

A correção cirúrgica de fraturas na diáfise femoral em crianças é frequentemente realizada com hastes flexíveis, especialmente na faixa etária de 5 a 11 anos e peso corporal inferior a 50 kg^4^. Esse método cirúrgico pode reduzir o tempo de hospitalização e o índice de complicações graves^1^.

Além disso, apesar das limitações inerentes ao presente tipo de estudo, como a impossibilidade de generalização de achados, é importante destacar que as complicações vasculares, apesar de raras, exigem vigilância clínica longitudinal e adequada. A formação de um pseudoaneurisma arterial como consequência tardia é ainda mais incomum, pois a literatura médica descreve poucos relatos parecidos, que geralmente são associados a problemas como migração do implante, posicionamento inadequado do material cirúrgico ou desgaste vascular lento e progressivo causado por fricção^8^.

É importante ressaltar ainda que o pseudoaneurisma de artéria femoral esquerda representa uma séria ameaça à função do membro, podendo causar compressão de nervos e músculos, trombose ou até mesmo uma ruptura exsanguinante. A abordagem terapêutica cirúrgica foi a mais adequada neste caso, diante da presença de manifestações clínicas progressivas, do risco de complicações isquêmicas e da confirmação de imagem aneurismática. A reconstrução vascular com ponte femoro-femoral, utilizando veia safena magna ipsilateral reversa, mostrou-se uma técnica eficaz^9^.

Este caso também alerta sobre a forma que complicações vasculares devem ser monitoradas como potenciais consequências de osteossínteses de fraturas em cirurgias ortopédicas, inclusive em pacientes pediátricos. Faz-se necessário seguimento adequado e com ampla investigação para exclusão de tais complicações potencialmente graves.

O presente estudo obteve autorização do Comitê de Ética e Pesquisa, sob o número de parecer 7.492.055 e segue os requisitos dispostos na Resolução 466/2012 para proteção de dados e preservação da identidade do caso relatado.

## CONCLUSÃO

A incidência de complicações vasculares relacionadas a osteossínteses é bastante rara, porém, o caso ilustrado de pseudoaneurisma arterial com surgimento 5 anos após procedimento cirúrgico em paciente pediátrico denota a importância de um seguimento longitudinal adequado, com suspeição clínica, uso de diagnóstico por imagem e atuação compartilhada entre cirurgiões ortopédico e vascular para identificação precoce de eventos vasculares que, apesar de escassos, podem ser clinicamente significativos.

## Data Availability

The data supporting this study are available upon request to the corresponding author, GBP, to ensure participant confidentiality.
